# OntoSecAI: Ontology-driven security automation for AI-enabled systems

**DOI:** 10.1371/journal.pone.0337806

**Published:** 2025-12-18

**Authors:** Ubaid Ullah, Muhammad Haleem, Asad Ullah

**Affiliations:** 1 IMT School for Advanced Studies, Lucca, Italy; 2 Department of Computer Science, Kardan University, Kabul, Afghanistan; 3 School of Information Engineering, Xi’an Eurasia University, Xi’an, China; Maulana Abul Kalam Azad University of Technology West Bengal, INDIA

## Abstract

The advent of artificial intelligence (AI) models presents significant opportunities alongside inherent security risks, such as the exploitation by adversaries generating malicious data to compromise other AI-enabled systems. Despite the urgent need to address such threats, AI-based threat modelling remains largely underexplored in research, primarily constrained by three key challenges: (i) the lack of formal representation of security and AI-based data, (ii) the absence of inference rules for automated threat identification, and (iii) inconsistent risk and vulnerability assessment. As a result, these limitations, coupled with stakeholders’ insufficient security knowledge and AI expertise, lead to erroneous threat modelling of AI-enabled systems. This research aims to develop and implement OntoSecAI, an ontology-based approach to automate threat modelling and assessment for AI-enabled systems. In particular, we design 03 ontologies and 30 inference rules, followed by risk and CVSS-based vulnerability assessments to perform automated threat modelling and assessment comprehensively. In addition, the approach is validated through 10 case studies and verified using mathematical theorems to confirm its correctness and completeness. The research findings demonstrate that the developed ontologies effectively facilitate unified representation and comprehensive coverage of security and AI systems’ data. Furthermore, the inference rules implemented effectively map system assets to potential security threats. Crucially, the utilization of ontologies provides consistent risk and vulnerability assessments across AI-enabled systems. Consequently, a comprehensive security knowledge base is offered to stakeholders, regardless of their varying security and AI expertise, ensuring uniform threat modelling across diverse AI systems and adaptability to emerging security threats.

## 1 Introduction

In recent years, artificial intelligence (AI) has emerged as a transformative technology, with its applications extending across diverse fields such as healthcare, finance, and education [1]. It also plays a central role on social media platforms [2], where AI algorithms are used to recommend personalized content, target advertising, and detect malicious activities [3]. This adoption of AI technologies is clearly illustrated by the rapid rise of platforms such as ChatGPT [4], which accumulated 100 million active monthly users in a short period, underscoring its broad appeal and value [5]. However, this widespread use of AI systems also faces significant security challenges [6,3]. Studies, such as those examined under MITRE ATLAS [7], show that AI systems are largely vulnerable to security threats by adversaries. Specifically, these threats exhibit a range of characteristics, encompassing (1) diverse attack vectors such as evasion, poisoning, and model replication [8,9]; (2) susceptibility in various deployment environments, including cloud [10], and edge-hosted machine learning models [11]; and (3) applicability to a wide range of use cases, notably attacks targeting AI systems like chatbots [3]. This scenario arises from the proliferation of readily accessible AI technologies, notably large language models (LLMs), among individuals and corporations, which amplified the potential to target other AI-enabled systems [6,12]. Examining this amplified scenario, a study [5] investigated the consequences of LLMs within the Swiss cybersecurity context, identifying threats such as spear phishing, vulnerable code injections, and remote code execution. This accessibility-driven expansion of AI underscores the increasing challenges in securing interconnected AI ecosystems. Based on this understanding, further studies, including those examined by OWASP [13], reveal that AI-enabled systems and models are highly susceptible to numerous security vulnerabilities. This susceptibility is further underscored by the fact that additional research highlights similar vulnerabilities in other AI technologies, including machine learning [14], federated learning [15], and computer vision [16], which can be exploited by adversaries.

Given the widespread vulnerability of AI systems to various threats, there is a critical need for effective security measures specifically tailored to AI systems [12]. One such measure is threat modelling, a proven technique in computer security that offers a valuable method for addressing the security challenges faced by AI systems [17]. Although recent studies have explored how to apply threat modelling specifically to AI [18,19,20,21], these efforts lack a broad coverage of security and AI systems data, which reduces the effectiveness of threat modelling as a tool for a wider assessment of AI security.

Furthermore, at present, supported solutions for threat modelling pose the following challenges: The primary challenge lies in the lack of formal data and conceptual models to support the fundamental representation of security aspects and AI systems. Conceptual models serve as a structured foundation defining how system data should be presented. The absence of clearly defined rules for inference hampers the reasoning process during threat identification. Inference rules guide how conclusions are drawn from available information. There is a gap in the definition of security threats specific to AI-enabled systems. Security threats need to be precisely described for effective threat modelling. The absence of consistent risk assessments for identified threats means that the severity and potential impact of each threat remain unclear. Moreover, without a clear understanding of the associated vulnerability assessment, prioritizing and addressing vulnerabilities becomes challenging. As a result of these limitations, different stakeholders may struggle to perform threat modelling and assessment effectively. This challenge is particularly pronounced for those with limited exposure to AI-related information, little cybersecurity expertise, or insufficient domain knowledge, ultimately reducing the overall security posture of AI systems. Hence, these gaps underscore the need for continued research on AI-specific security to keep pace with AI’s evolving threat landscape, as the importance of securing AI-enabled systems against emerging threats has become increasingly critical [6,22].

To fill these gaps, the main objective of this study is to design and implement OntoSecAI, an ontology-driven approach for the automated threat modelling and assessment of AI-enabled systems. To achieve the main objective, we pursue the following specific objectives: (1) to construct a holistic ontology that comprehensively models the lifecycle of the AI system and its unique security characteristics, thereby establishing a unified security knowledge base for AI-enabled systems; (2) to design and implement a set of robust inference rules that enable automated threat identification; (3) to conduct risk and vulnerability assessments aligned with threat modelling practices; and (4) to verify and validate the correctness and completeness of the proposed approach through formal mathematical proofs and across diverse case studies. These objectives collectively structure the research contributions as their outcomes.

In this context, the ontology provides a structured way to organize and represent the entities of the system and their relationships, allowing the knowledge base to be consistently interpreted and applied across different AI-enabled systems in the security domain [23,24,25].

In summary, our *contribution* is fivefold:

1. We designed a *domain ontology* to comprehensively describe entire AI-enabled systems, overcoming domain-specific challenges through a formal and shared vocabulary for all stakeholders.
2. We developed a *unified knowledge base* for general AI-enabled systems by including diverse assets, security threats, system vulnerabilities, adversarial tactics and techniques, AI system lifecycle, and mitigations from multiple data sources.
3. We provided a well-defined *rule set* to characterize threat modelling logic and accurately map AI entities to potential security threats. This ensures accuracy in mapping the system to potential security threats.
4. We performed a consistent *risk assessment* to understand the severity of defined threats. In addition, we performed a *vulnerability assessment* to help define the severity of the vulnerabilities.
5. We *validated* our methodology using 10 case studies and *verified* its correctness and completeness using mathematical theorems. This rigorous validation and verification process provides strong evidence for the reliability and trustworthiness of our automated AI threat modelling methodology.

Beyond its contributions, our approach also faces two limitations that define its scope. Since the cybersecurity landscape continuously evolves with new threats and vulnerabilities, maintaining the ontology is a challenging and ongoing task. Furthermore, scalability must be considered; as the knowledge base expands, reasoning demands may increase significantly, potentially hindering real-time analysis in resource-constrained environments.

The *organization* of this paper is as follows: Sect 2 discusses related work, which encompasses a review of existing approaches covering AI-centric threat modelling, ontology-driven threat modelling, and risk and vulnerability assessments. Sect 3 presents the research design for automated threat modelling and assessment using ontologies. In Sect 4, the validation and verification of the approach are performed. In Sect 5, we identify potential threats to validity and how to deter these threats. Sect 6 presents the conclusion of our work and outlines future directions.

## 2 Related work

This study builds on existing work in three important areas: (i) AI-centric methodologies, focusing on AI-based threat modelling; (ii) ontology-centric methodologies, focusing on ontology-based threat modelling in various areas; and (iii) risk and vulnerability assessment. To provide a foundational basis for our study, we performed a literature review using *Google Scholar* and *IEEE Xplore*, widely recognized sources for academic research. We applied the search strings *“ontology-driven threat modelling”*, *“AI-driven threat modelling”*, and *“risk and vulnerability assessment”*. From the retrieved works, we applied a strict relevance criterion: only studies that directly addressed our research scope are included, while less relevant studies are excluded. This method ensured that only contributions closely aligned with our objectives were considered. The most relevant findings from previous studies are summarized in the following sections.

### 2.1 AI-centric threat modelling

Wang et al. [14] reviewed the security challenges and recent advances in AI research. They categorized security threats at each stage, including (1) sensor spoofing and scaling attacks during data preprocessing and (2) poisoning and adversarial attacks during training and inference. Their work provides a comprehensive overview of the AI security landscape. However, their study does not address the novel threats and mitigation strategies that have emerged with the advent of LLMs. Similarly, Hu et al. [26] examined recent advances throughout the AI lifecycle, from data collection to deployment, and provided an overview of the AI security landscape. The authors summarized relevant countermeasures and discussed challenges and opportunities in securing AI systems. Although the study provides a useful overview of AI security throughout the lifecycle, it relies on outdated threats and mitigations, overlooking risks from emerging technologies such as LLMs. It also omits system weaknesses, risk and vulnerability assessments, and evolving adversarial tactics.

Guembea et al. [27] investigated the emerging dangers of AI-powered cyberattacks. Their findings revealed that a significant portion of AI-driven attacks occur during the access and penetration phase of the cyber kill chain. Their investigations highlight the potential inadequacy of existing cyber defenses against the increasing complexity of these attacks, emphasizing the need to invest in AI cybersecurity infrastructures.

To evolve the AI threat landscape, Assen et al. [18] introduced ThreatFinderAI, an approach to model AI-related assets, threats, countermeasures, and residual risk quantification. To evaluate its practicality, the approach is validated through an AI-based healthcare platform. In addition, the tool was used to identify and discuss strategic risks in an LLM-based application through a case study. In parallel, Hoseini et al. [28] proposed a structured approach to threat modelling for AI and machine learning (ML) systems using attack trees and a risk analysis method. It outlines a multistep procedure for designing and evaluating threat models and classifying attacker goals and attack outcomes based on security violations. However, their attack tree model is a static representation of threats. Their work has a limited scope, as it excludes key attack details, assumes a centralized learning environment, and does not address the unique security challenges of non-centralized scenarios like federated learning. Our approach is designed with the flexibility to handle such complex systems.

To further extend the threat landscape of AI, Das et al. [29] provided a review of the security and privacy challenges associated with AI models. The authors examined vulnerabilities in both training data and user interactions, discussed application-based risks across various domains, and reviewed potential defense mechanisms against these emerging threats. However, its main limitations are the scope and the absence of a concrete framework. Although it offers a useful overview of LLM security, its survey-based nature lacks a specific and implementable solution. This contrasts with our work, which develops a novel ontology-driven solution. In a related effort to address the security concerns of AI, Mauri et al. [30] addressed the risks associated with AI’s increasing application in socio-technical infrastructures, particularly focusing on the unique vulnerabilities of machine learning (ML). The authors proposed STRIDE-AI, a methodology that aims to guide ML practitioners in selecting effective security controls, illustrated with a real-world use case. The primary limitations are its reliance on a manual process, a static and limited scope, and a lack of integrated assessment. Unlike STRIDE-AI, our ontology is automated, extensible, and capable of integrating risk analysis for a more comprehensive security view.

### 2.2 Ontology-centric threat modelling

Preuveneers et al. [21] proposed an ontology-based framework specifically designed for AI-enabled systems to model prevalent threats and countermeasures. However, its selective threat coverage and lack of explicit automation rules contrast with our research. We offer a more comprehensive representation of cybersecurity knowledge, including various threats, weaknesses, and mitigations, along with a formalized set of rules for automated threat identification targeting AI system assets, a feature absent from their approach.

Similarly, Kougioumtzidou et al. [31] presented an AI-assisted framework to build and update cybersecurity taxonomies and ontologies. For ontology construction, they propose a conceptual schema based on the STIX 2.1 standard and utilize the Owlready2 Python library. Their framework is limited by domain specificity, as the paper’s core technology is a language model fine-tuned for the cybersecurity domain and is limited to knowledge only encoded in text. In contrast, our approach enables broader formal reasoning, comprehensive threat modelling, and coverage of a wide range of cybersecurity knowledge and related assessments.

Manzoor et al. [32] addressed the challenges of complex cloud threat analysis through the development of a dedicated ontology. This ontology comprehensively models actors in the cloud, their requirements, interactions between cloud services, and potential vulnerabilities that could violate these requirements. By mapping this ontology to a design structure matrix, their approach facilitates security assessments from various actor viewpoints.

Another contribution comes from Kamongi et al. [33], who introduced Nemesis, an automated architecture for cloud threat modelling and risk assessment. They utilized ontologies as knowledge bases to model threats and assess risks in cloud systems. Their approach built ontologies for vulnerabilities, defenses, and attacks, automatically instantiating them into Ontology Knowledge Bases (OKBs) that capture relationships between these elements.

Salini et al. [34] proposed an ontology-based system to predict and classify web application attacks. Their system effectively stores information about threats, vulnerabilities, and attacks, enabling the prediction of attacks by analyzing the relationships between threats and vulnerabilities. Attacks are classified according to their severity regarding security goals. Furthermore, the system offers suggestions for prevention and countermeasures in order to assist developers in building more secure web applications. In contrast, Tok et al. [35] proposed SCOPE to address cybercrime and digital forensics in Smart City Infrastructures (SCI). Recognizing limitations in existing tools and ontologies regarding information sharing and interoperability, SCOPE integrates SCI threat models, digital forensic evidence, and MITRE attack information. They showcased SCOPE’s ability to represent complex SCI-specific threats and investigation workflows using real-world APT incident scenarios, making it available for community-based identification and sharing of cyber threats in emerging SCI trends.

Similarly, Välja et al. [36] addressed the complexity and resource demands of threat modelling by proposing an ontology framework to improve automation. They highlighted the issue of lacking context in automatically collected data and suggested using ontologies to inject domain knowledge. The framework aimed to simplify the creation of the model by standardizing the input sources, eliminating duplicates, and logically grouping software.

Luh et al. [37] presented TAON, an OWL-based ontology designed to mitigate advanced persistent threats (APTs). TAON provides a holistic view of actors, assets, and threat details, mapping them to detectable events and anomalies. The ontology aims to facilitate the development of behavior-based detection systems and offers a means for organizations to plan defenses against APTs by understanding the "how, why, and by whom" of targeted attacks. Populated with data, TAON becomes a smart correlation framework for semantic assessment. In contrast, Sabbagh et al. [38] proposed a socio-technical framework for the modelling of threats within the global software supply chain, with their approach validated through a case study of the Swedish Armed Forces. Their framework addresses modelling the target system, identifying threats within the complex socio-technical landscape of the supply chain, and analyzing potential countermeasures. Finally, Rosa et al. [24] presented ThreMA, an approach to automate threat modelling for ICT infrastructures using ontologies. They highlighted the challenges of manual threat modelling and the need for standardized representations and automated reasoning. ThreMA provides a formal vocabulary for modelling ICT infrastructure, a threat catalog, and inference rules for threat identification. The approach was validated through case studies from the Italian public sector.

### 2.3 Risk and vulnerability assessment

Maunero et al. [39] proposed an ontology-based approach to automate the risk assessment process for ICT infrastructures. Their work focuses on creating a structured and formal representation of the descriptions of the ICT infrastructure and related security information using a defined ontology. The ontology follows an asset-oriented approach, linking infrastructure components with security data to improve automation. In contrast, Phillips et al. [40] introduced a simulation-based method for the automated assessment of the risk of cyberphysical systems (ISO 27005). Modelling threat cause-and-effect and system interdependencies, it uses a knowledge base to simulate attacks and calculate risk based on controls. Implemented in Spyderisk, it was validated with a steel mill attack case study.

Furthermore, Arora et al. [41] examined risk and vulnerability assessment techniques for the automotive industry due to increased cybercrime threats with wireless car connections and advancements in autonomous driving. The study highlights the potential for significant damage from vehicle breaches and the importance of enhancing vehicle security.

### 2.4 Research summary

Table 1 presents a conceptual comparison of existing threat modelling studies, evaluated against *15 key coverage criteria*. The AI-centric studies generally fail to address many of these criteria, while the remaining 12 non-AI-focused studies also exhibit significant gaps and limitations. In the table, a checkmark (✓) indicates that the criterion is addressed, whereas a dash (–) denotes a lack of coverage. However, it should be noted that a checkmark may represent only a *partial fulfillment* of the criterion, rather than a comprehensive one. Furthermore, analysis of existing studies, including those beyond the related work, reveals several other critical limitations. These studies present a *constrained scope*, as their coverage of *threats, countermeasures, and weaknesses* is outdated or highly limited, thereby failing to address the vulnerabilities of modern AI systems and technologies. A *significant gap* is the absence of standard *vulnerability assessment*, with the few studies that attempt it using outdated methodologies and failing to adopt standardized systems like CVSS. In addition, there is a notable lack of comprehensive *inference rules* and a complete absence of *verification* for the proposed approaches. *Mitigation strategies* are often absent throughout the AI lifecycle, and even in studies that address them, the coverage of the complete *AI lifecycle* phases is consistently incomplete. Key aspects of *adversarial modelling*, including adversaries, their activities, tactics, and techniques, have either been only partially addressed or completely overlooked. Hence, all observed deficiencies underscore the critical need for a robust approach to address these identified gaps.

**Table 1:** Conceptual comparison of existing studies.

Criterion	[14]	[18]	[21]	[26]	[27]	[28]	[29]	[30]	[24]	[31]	[32]	[33]	[34]	[35]	[36]	[37]	[38]	[39]	[40]	[41]
AI-Centric Focus	✓	✓	✓	✓	✓	✓	✓	✓	–	–	–	–	–	–	–	–	–	–	–	–
Lifecycle Coverage	✓	✓	✓	✓	–	✓	–	✓	–	–	–	–	–	–	–	–	–	–	–	–
Ontology-Driven Modelling	–	–	✓	–	–	–	–	–	✓	✓	✓	✓	✓	✓	✓	✓	✓	✓	✓	–
Threat Coverage	✓	✓	✓	✓	✓	✓	✓	✓	✓	✓	✓	✓	✓	✓	✓	✓	✓	✓	✓	✓
Weakness Coverage	–	–	✓	✓	✓	✓	–	✓	–	✓	✓	✓	✓	✓	✓	–	✓	–	–	–
Mitigation Coverage	✓	✓	✓	✓	–	–	✓	✓	✓	–	–	✓	✓	–	–	✓	–	–	–	✓
Risk Assessment	–	✓	–	–	–	✓	–	✓	–	–	–	✓	–	✓	✓	✓	–	✓	✓	✓
Vulnerability Assessment	–	–	✓	–	–	–	–	–	–	–	–	✓	–	–	✓	–	–	–	–	–
Adaptability to New Threats	–	–	✓	–	–	–	–	–	✓	✓	–	✓	–	–	✓	–	–	✓	✓	✓
Asset Coverage	–	✓	✓	✓	–	✓	–	✓	✓	–	–	✓	–	✓	✓	✓	–	–	✓	✓
Inference Rule Provision	–	–	–	–	–	–	–	–	✓	–	–	–	✓	✓	✓	✓	–	–	–	–
Adversary Modelling	–	–	✓	–	✓	✓	–	✓	✓	–	–	–	–	–	–	✓	–	–	–	–
Validation Method	–	✓	✓	–	–	✓	–	✓	✓	–	–	✓	✓	✓	✓	–	✓	✓	✓	✓
Verification Method	–	–	–	–	–	–	–	–	–	–	–	–	–	–	–	–	–	–	–	–
Implementation Status	–	✓	✓	–	–	✓	–	–	✓	✓	✓	✓	✓	✓	✓	✓	–	✓	✓	✓

To address these *research gaps and limitations*, this study introduces a formally verified, *ontology-driven approach* for the automated threat modelling and assessment of AI-enabled systems. The proposed approach designs a comprehensive domain ontology for AI systems and establishes precise rule-based threat mappings to support systematic analysis. The inference rules that map system assets to their potential security threats are formally defined using the Semantic Web Rule Language (SWRL). OntoUML is adopted as the conceptual modelling language due to its formal ontological foundation, which ensures semantic consistency across AI security concepts. The ontology is implemented in Protégé, serving as the primary development environment, and incorporates risk and vulnerability assessments directly within the threat modelling process to achieve an integrated and coherent representation of AI systems security. In contrast to prior studies, which exhibit limited coverage across multiple criteria, the proposed approach provides comprehensive coverage across the entire threat modelling spectrum. It explicitly integrates security threats, weaknesses, and mitigations coverage and enables adversaries and assets modelling. This approach effectively addresses the fragmented and narrowly scoped nature of existing studies, many of which lack adaptability to emerging AI threats or fail to incorporate formal validation mechanisms.

## 3 Research design

This study aims to automate threat modelling for AI-enabled systems using ontologies. To achieve this aim, we are required to (i) build a comprehensive ontology using formal data, (ii) generate inference rules to map security threats to the corresponding system assets correctly, and (iii) calculate risk and vulnerability to provide a consistent assessment across AI systems. Consequently, we formulate the following research questions *(RQ*_*s*_): to guide our investigation:

- ***RQ***_***1***_ ***: How can a comprehensive ontology be constructed to represent AI-enabled systems and their security aspects to enable effective automated threat modelling?***
  The purpose of *RQ*_*1*_ is to construct a comprehensive domain ontology that captures AI-enabled systems and their security aspects, forming a unified knowledge base that serves as the foundation for effective automated threat modelling. The purpose frames the constructive task (how to build it), domain scope (AI-enabled systems and their security aspects), and intended outcome (effective automated threat modelling).
- ***RQ***_***2***_ ***: How effective are inference rules in accurately identifying potential threats to system assets in AI-enabled systems?***
  The purpose of *RQ*_*2*_ is to assess how well the inference rules can accurately detect potential threats to assets in AI systems.
- ***RQ***_***3***_ ***: How can a risk and vulnerability assessment be effectively conducted for AI-enabled systems in alignment with threat modelling practices?***
  The purpose of *RQ*_*3*_ is to perform risk assessments of identified threats and vulnerability assessments of system weaknesses in AI-enabled systems to support effective security modelling.

Collectively, these *RQ*_*s*_ establish an interconnected flow: we begin by constructing ontologies *(RQ*_*1*_), then define inference rules to automate threat identification *(RQ*_*2*_), and conclude by performing the risk and vulnerability assessment *(RQ*_*3*_).

### 3.1 Proposed approach

**Fig 1:**
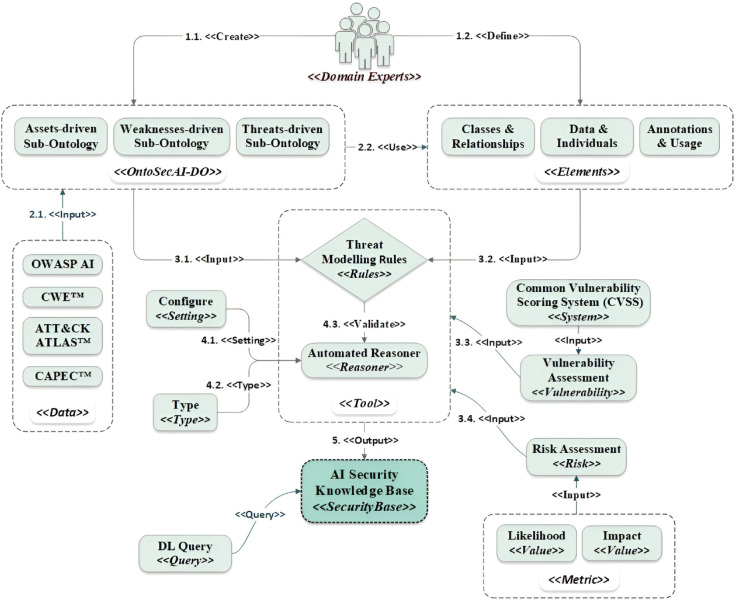
OntoSecAI approach.

This section introduces the proposed approach,*OntoSecAI*, which provides a structured and automated process for AI security threat modelling and assessment. Automation in the approach encompasses three core capabilities: (i) automated identification of security threats through inference rules; (ii) automated risk and vulnerability assessment based on defined attributes; and (iii) automated generation of reports summarizing identified threats, vulnerabilities, and mitigations. The approach is supported by a formally defined ontology, *OntoSecAI-DO* (Domain Ontology), which encapsulates the conceptual knowledge essential for enabling the process. Fig 1 depicts the high-level conceptual workflow through which domain experts construct the domain ontology using predefined ontological elements.

OntoSecAI-DO consists of three sub-ontologies, i.e., an assets-driven sub-ontology, a weaknesses-driven sub-ontology, and a threats-driven sub-ontology, each representing a distinct yet interrelated dimension of AI-enabled system security. The assets-driven sub-ontology focuses on the system assets, their operational aspects, and structural dependencies. The weaknesses-driven sub-ontology specifically focuses on the known vulnerabilities within the AI system’s infrastructure, such as network insecurities or flawed data management practices. The threats-driven sub-ontology encompasses a wide range of tactics and techniques, security threats, and mitigation mechanisms. These sub-ontologies are discussed in Sect 3.3. These ontologies consist of classes interconnected by relationship properties. The data property is used to instantiate data instances, while the individual property is utilized to create object instances for the ontologies. Similarly, the annotation property describes the ontologies, providing essential context. In contrast, the usage property enables stakeholders to visualize how specific ontology elements, such as classes, properties, or individuals, are utilized, facilitating effective management and maintenance of the ontology. Furthermore, the approach incorporates risk and vulnerability assessments, which are calculated using the risk scoring model and the Common Vulnerability Scoring System (CVSS) v3.1 metrics [42], respectively. The Semantic Web Rule Language (SWRL) [43] specifies the inference rules that map system assets to the security threats they may encounter. The automated reasoner, configured with specific settings and types, validates these rules to generate a comprehensive security knowledge base for all AI-based systems. Each of these components plays a critical role in automating ontology-driven threat modelling.

Protégé [44] serves as the collaboration platform, facilitating the creation and maintenance of these ontologies and rules. This tool is also used in an ontology-driven approach to automate threat modelling for ICT infrastructure [24]. Furthermore, built-in features such as OWL provide the foundational language for constructing ontologies that capture intricate domain knowledge. The axioms within these ontologies define essential logical constraints, ensure data consistency, and accurately classify entities. Automated reasoners (e.g., Pellet, HermiT) process axioms and SWRL rules to generate a security knowledge base. To save the knowledge base, the tool offers several format choices, with RDF/XML being a widely used and well-supported standard. We can use the description logic (DL) feature to retrieve ontology knowledge using expressions based on defined relationships.

### 3.2 Data collection and validation

In this study, the data is collected from four widely recognized cybersecurity repositories: OWASP AI [13], CWE [45], MITRE ATLAS [7], and CAPEC [46], which provide highly comprehensive data that form the foundation of the ontological structure. These repositories are chosen because they provide community-validated data, which makes them highly relevant to AI threat modelling. Data are extracted from OWASP AI and MITRE ATLAS through a semi-automated process, since both repositories provide structured and downloadable formats. In contrast, data from CWE and CAPEC required manual curation to ensure contextual relevance and proper mapping to specific threats. This hybrid approach ensured that only AI-relevant data is included. Table 2 presents the complete statistics of the ontological data, including classes and their corresponding instances.

**Table 2:** Ontology statistics.

No.	Main Class	Instances
01	Asset	87
02	Weakness	55
03	Security Threat	33
04	Mitigation	80
05	Tactic	14
06	Technique	57
07	Adversary	06
08	Adversary Activity	11
09	AI Lifecycle Stage	10
10	CIA	03
11	Risk	02

To ensure the data compliance with our approach, it is validated through a two-step process: (1) collected data are cross-checked against definitions provided in the repositories, ensuring consistency with community-accepted standards; (2) class definitions and their mappings are reviewed through an expert validation exercise involving two domain experts in cybersecurity and AI, who verified that the classes and relationships are relevant, correctly classified, and semantically consistent. This process ensured that the ontology not only reflected widely recognized terminologies but also maintained accuracy for AI threat modelling.

### 3.3 Ontology design

To design the domain ontology using a standard conceptual language, we adopted OntoUML [47], a modelling language that extends Unified Modelling Language (UML) with a formal ontological foundation to ensure semantic soundness. Unlike standard UML, OntoUML distinguishes classes through specific stereotypes (e.g., <<kind>>, <<quantity>>), providing ontological clarity beyond object-oriented abstractions. It also supports precise relationships, including part–whole associations (aggregation, composition) and generalizations, enabling precise representation of complex domains. Cardinality in diagrams specifies the number of instances allowed in a relationship, thereby ensuring a precise and unambiguous interpretation (for example, 1 denotes exactly one instance, while 1..* indicates one or more).

Table 3 defines 29 core ontology classes, which capture adversarial behavior (e.g., Tactics, Techniques, Adversary, Threats), system assets (e.g., Software, Data, Platform, etc.), system properties and vulnerabilities (e.g., CIA, Weaknesses, Risks), and defensive mechanisms (e.g., Mitigations, Security Mechanisms). Table 4 summarizes the 18 relationships connecting the ontology classes.

**Table 3:** Ontology classes.

No.	Class	Description
01	<<System>>	An AI-enabled system that can be targeted by adversaries and is exposed to threats.
02	<<Asset>>	A component of a system that may possess a weakness and is subject to a threat.
03	<<Platform>>	A type of asset representing the underlying platform within a system.
04	<<Resource>>	A type of asset representing a supporting resource in a system.
05	<<Hardware>>	A type of asset representing a physical component of a system.
06	<<Software>>	A type of asset representing a software component in a system.
07	<<Data>>	A type of asset comprising a dataset and information processed within the system.
08	<<Network>>	A type of asset representing the networking infrastructure that supports the system’s operation.
09	<<Tactic>>	A high-level strategic goal employed by an adversary to conduct malicious activity. Each tactic consists of one or more techniques.
10	<<Technique>>	A specific method or action used by an adversary to achieve a tactic. A technique contributes to the realization of a security threat.
11	<<Activity>>	An action performed by an adversary with the intent to compromise a system.
12	<<Threat>>	A potential danger or risk arising from an adversarial technique, which can violate CIA properties and be addressed by mitigation.
13	<<Adversary>>	An entity that executes a malicious activity using a tactic and a technique.
14	<<Lifecycle>>	The phases of AI system development and deployment, within which a relevant mitigation can be applied.
15	<<Property>>	A fundamental security property — Confidentiality, Integrity, and Availability — that may be violated by a threat.
16	<<Mitigation>>	A safeguard or control designed to prevent a technique or mitigate the impact of a threat. It is embedded within the AI lifecycle.
17	<<Mechanism>>	A defensive measure or control implemented to protect an asset from a threat.
18	<<Risk>>	The likelihood and potential impact of loss or damage, arising from a weakness and manifested through a threat.
19	<<Weaknesses>>	A vulnerability or flaw that introduces risk and is potentially exploited by a threat.
20	<<Software Weaknesses>>	A subclass of weakness comprising an algorithm weakness, a model weakness, and a data weakness.
21	<<Organizational Weaknesses>>	A subclass of weakness arising from a human-centric factor and a policy or procedure. This weakness is a logical composition, where a specific human-centric factor and a policies & procedures weakness must both be present to form the organizational weakness.
22	<<Human-centric Factors>>	An organizational weakness arising from human behavior, error, or bias. This is the specific human action, such as a developer’s error in handling user inputs or a data scientist’s cognitive bias in data labeling.
23	<<Policies & Procedures>>	An organizational weakness stemming from inadequate or ineffective governance, rule, or process. This is the systemic flaw that allows the human factor to persist, such as an inadequate data validation process or a lack of a clear model update policy.
24	<<Hardware Weaknesses>>	A subclass of weakness associated with a vulnerability in a physical component.
25	<<Network Weaknesses>>	A subclass of weakness associated with a flaw in network infrastructure.
26	<<Infrastructure Weaknesses>>	A subclass of weakness related to a vulnerability in the underlying computing infrastructure.
27	<<Algorithm Weakness>>	A software weakness associated with a flaw in algorithmic design.
28	<<Model Weakness>>	A software weakness related to a vulnerability in a machine learning model.
29	<<Data Weakness>>	A software weakness related to the integrity, quality, or availability of data.

**Table 4:** Ontology relationships.

No.	Relationship	Description
01	<<Cause>>	Indicates that one class is the direct cause of another.
02	<<ComposedOf>>	Denotes that a class is made up of other classes.
03	<<ConsistOf>>	Shows that a class is comprised of other classes.
04	<<IsThreatenedBy>>	Represents that one class is under threat from another.
05	<<ProtectsFrom>>	Indicates that a protective measure guards against a threat.
06	<<IsProtectedBy>>	Represents that an asset is secured by a mechanism.
07	<<Exploits>>	Shows that one class takes advantage of another.
08	<<Has>>	Indicates that a class possesses a certain characteristic or weakness.
09	<<Impact>>	Shows that one class affects another.
10	<<Violates>>	Represents that a threat breaches a property.
11	<<LeadsTo>>	Indicates that one action or item results in another.
12	<<IsPreventedBy>>	Shows that a technique can be stopped by a mitigation.
13	<<IsIncludedIn>>	Denotes that an item is part of a larger life cycle.
14	<<IsMitigatedBy>>	Represents that a threat is reduced or lessened by a mitigation.
15	<<InitiatedBy>>	Shows that an action or threat originates from an entity.
16	<<Uses>>	Indicates that one class employs another.
17	<<Performs>>	Represents that an entity carries out an activity.
18	<<IsA>>	Indicates that a class is a specialization or a type of another class

The main ontology consists of three sub-ontologies. The implementation of these ontologies involves the conversion of conceptual models into a functional ontology. This process, guided by our approach, ensures that all relationships and logical constraints are instantiated accurately, making the ontology ready for automated reasoning. The conceptual designs and compositions of the three proposed ontologies are presented as follows:

#### 3.3.1 Assets-driven sub-ontology.

**Fig 2:**
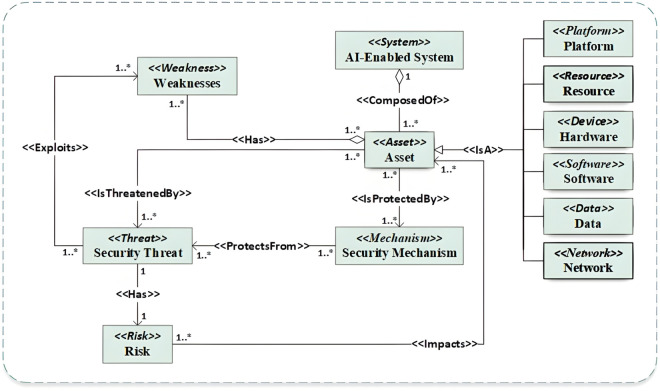
Assets-driven sub-ontology.

Fig 2 illustrates the conceptual model designed to create an assets-driven sub-ontology, representing AI systems and their associated assets. Due to the increasing vulnerability of these assets to various attacks [48], the ontology emphasizes effective asset management and threat identification. The development of this ontology underscores the need to consider a wide range of assets, encompassing software and hardware components, as well as network communications. Within the model, relationships are carefully defined to show how assets are related to other classes, such as security threats and inherent weaknesses.

#### 3.3.2 Weaknesses-driven sub-ontology.

**Fig 3:**
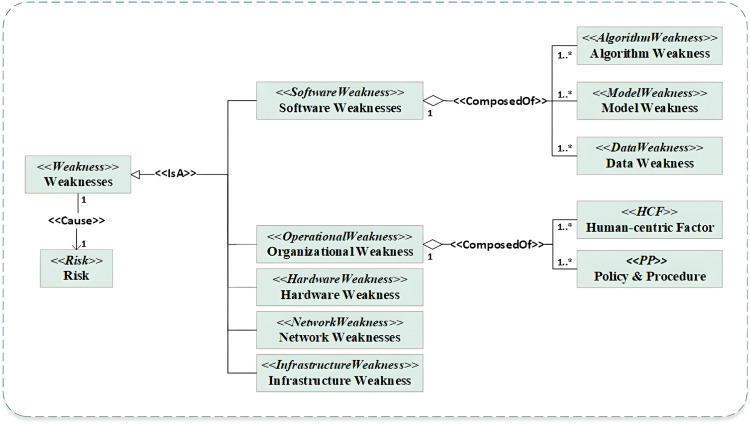
Weaknesses-driven sub-ontology.

The weaknesses-driven sub-ontology forms a key component of the main ontology, designed to enhance the modelling of weaknesses in AI-enabled systems. As illustrated in Fig 3, its conceptual model specifically structures known weaknesses and vulnerabilities associated with AI systems. The vulnerabilities are derived from two primary sources: OWASP AI and CWE, which provide a comprehensive overview of potential security weaknesses. By systematically organizing these weaknesses, the sub-ontology enables security professionals to identify and prioritize the most critical weaknesses requiring attention.

#### 3.3.3 Threats-driven sub-ontology.

**Fig 4:**
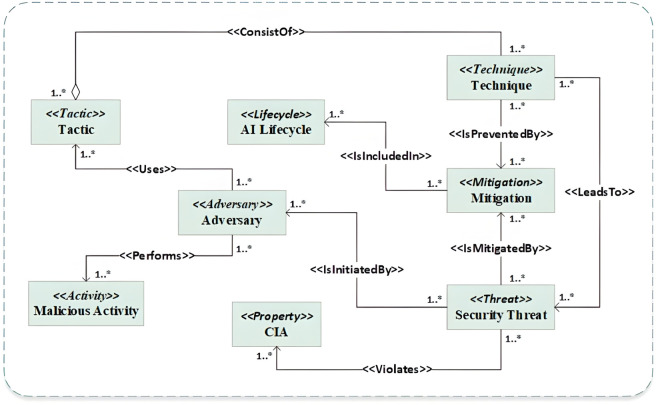
Threats-driven sub-ontology.

The threats-driven conceptual model, as shown in Fig 4, is employed for threats-driven sub-ontology. We extract security threats from the MITRE ATLAS and CAPEC, which offer a comprehensive catalog of tactics, techniques, and mitigations. Additionally, we incorporate mitigation strategies from OWASP AI and MITRE ATLAS to ensure a robust foundation for addressing these threats. Furthermore, we integrate the AI lifecycle into this ontology, enabling the mapping of mitigation mechanisms to the corresponding phases of the lifecycle to effectively prevent potential threats.

### 3.4 Threat modelling rules

Threat modelling automation in AI systems uses inference rules specified through the SWRL. These rules, processed by an automated reasoner, facilitate the mapping of security threats to the corresponding components of the system. SWRL rules consist of two components: the antecedent and the consequent. The antecedent defines the conditions that must be satisfied for the rule to apply, while the consequent specifies the actions to be executed if the antecedent conditions are met. This can be mathematically expressed as: If *P*, Then *Q*

P (Antecedent)⟹Q (Consequent) (1)

In the context of the rule P⟹Q, it asserts that if *P* is true, then *Q* must also be true. Consequently, the inference rules shown in Table 5 are derived to identify threats to assets. The specific relationships used in these rules are defined in Table 6. The first 19 relationships are used to express the antecedent, while the 20th relationship is used for the consequent, which is consistent with its definition in Table 4.

**Table 5:** Inference rules.

Rule No.	If [Condition], then [Threat]
Rule 1	If an *AI-based Library* is compromised during development, then the library is threatened by *Supply Chain Poisoning*.
Rule 2	If an *AI-enabled System* is vulnerable to arbitrary code execution, then the system is threatened by *Arbitrary Code Execution*.
Rule 3	If the *AI-based Source File or Repository* is misconfigured, then the source file or repository is threatened by *Misconfiguration of the Source File/Repository*.
Rule 4	If an *AI-enabled System* contains a backdoor and is detected by a backdoor detector, then the system is threatened by a *Backdoor Attack*.
Rule 5	If a *AI-based Resource* is hijacked by an adversary, then the resource is threatened by *Resource Hijacking*.
Rule 6	If an *AI Model* is replicated by an adversary, then the model is threatened by *Model Replication*.
Rule 7	If an *AI Chabot* is poisoned by an adversary, then the Chabot is threatened by *Chabot Poisoning*.
Rule 8	If an AI-enabled System is evaded by an adversary using authentication evasion techniques, then the system is threatened by *Authentication Evasion*.
Rule 9	If an *AI-enabled System* is injected with crafted prompts by an adversary, then the system is threatened by *Prompt Injection*.
Rule 10	If an adversary allocates excessive resources, then the *AI-based Resources* are threatened by *Excessive Allocation*.
Rule 11	If an *AI-based Algorithm* is exploited by an adversary to bypass detection, then the algorithm is threatened by *Bypassing Detection*.
Rule 12	If an *AI-enabled System* is bypassed by an adversary using authentication bypass techniques, then the system is threatened by *Bypassing Authentication*.
Rule 13	If an *AI-enabled System* is under a DoS attack by an adversary, then the system is threatened by a *Denial-of-service Attack*.
Rule 14	If a *Data* is exposed by an adversary, then the data is threatened by *Privacy Leaks*.
Rule 15	If a *Model or Network Traffic* is manipulated to evade detection by an adversary, then the model or network is threatened by *Detection Evasion*.
Rule 16	If a *GPT Model* is poisoned by an adversary, then the model is threatened by *GPT Poisoning*.
Rule 17	If a *Hardware’s Information* is deduced from alternative streams by an adversary, then the hardware is threatened by a *Side Channel Attack*.
Rule 18	If a *Hardware* is disrupted by faulty inputs, then the hardware is threatened by a *Fault Injection Attack*.
Rule 19	If a malicious backdoor is inserted into *Hardware* by an adversary, then the hardware is threatened by *Hardware Trojan Attack*.
Rule 20	If an adversary injects unauthorized resources into a *Network*, then the network resources are threatened by *Resource Injection*.
Rule 21	If an *AI-based System* is reverse-engineered by an adversary, then the system is threatened by an *Inversion Attack*.
Rule 22	If a *Model’s Code* is accessed by an adversary, then the model is threatened by *Model Theft*.
Rule 23	If a *Model’s Training Data* is manipulated by an adversary to expose sensitive information, then the model is threatened by *Membership Inference Attack*.
Rule 24	If a *Model’s Training Data* is manipulated by an adversary to behave in an undesirable way, then the model is threatened by *Model Skewing Attacks*.
Rule 25	If a *Model’s Output* is manipulated by an adversary, then the model is threatened by an *Output Integrity Attack*.
Rule 26	If an *AI-shared Resources*, such as GPUs, are manipulated by an adversary, then the AI resource is threatened by *Resource Manipulation*.
Rule 27	If an adversary inserts malicious logic into *Hardware*, then the hardware system is threatened by *Malicious Logic Insertion*.
Rule 28	If an adversary intercepts or alters *Communication* between two systems, then the communication channel is threatened by an *adversary-in-the-middle Attack*.
Rule 29	If an adversary manipulates the *Communication Protocols*, then the communication protocol is threatened by *Protocol Manipulation*.
Rule 30	If an adversary injects malicious data into *Network Traffic*, then the network traffic is threatened by *Traffic Injection*.

**Table 6:** Relationships used in inference rules.

No.	Relationship	Description
01	<<IsCompromisedBy>>	Represents that an asset has been compromised due to adversarial interference.
02	<<IsVulnerableTo>>	Represents that an asset has a weakness that can be exploited by a specific threat.
03	<<IsMisconfigured>>	Represents that an asset has been incorrectly configured, creating security risks.
04	<<ContainsBackdoor>>	Represents that an asset has an embedded malicious backdoor that can be exploited.
05	<<IsHijackedBy>>	Represents that an asset is taken over or controlled by an adversary.
06	<<IsReplicatedBy>>	Represents that a model is illegally copied or cloned by an adversary.
07	<<IsPoisonedBy>>	Represents that a system is manipulated with malicious data or inputs by an adversary.
08	<<IsEvadedBy>>	Represents that adversarial evasion techniques bypass a system’s defenses.
09	<<IsInjectedWith>>	Represents that an asset is compromised through the injection of prompts, malicious code, or crafted data.
10	<<IsExploitedBy>>	Represents that an algorithm or vulnerability is leveraged by an adversary to gain advantage (e.g., bypassing detection).
11	<<IsAttackedBy>>	Represents that an asset is directly targeted by an attack such as DoS, or side-channel exploitation.
12	<<IsExposedBy>>	Represents that sensitive data or resources are revealed, leaked, or made accessible to an adversary.
13	<<IsManipulatedBy>>	Represents that an asset (e.g., model) is altered by an adversary to achieve malicious outcomes.
14	<<IsDeducedFrom>>	Represents that information about hardware is inferred by adversaries via indirect means (e.g., side-channel analysis).
15	<<IsInjectedInto>>	Represents that adversaries insert malicious resources, code, or traffic into systems or networks.
16	<<IsReverseEngineeredBy>>	Represents that a system is analyzed or deconstructed by an adversary to reveal its design, vulnerabilities, or parameters.
17	<<IsAccessedBy>>	Represents that unauthorized entities gain access to sensitive assets, such as a model’s code or training data.
18	<<HasInsertedLogic>>	Represents that malicious logic is deliberately inserted into hardware or software.
19	<<IsInterceptedBy>>	Represents that communication channels are captured, altered, or monitored by an adversary.
20	<<IsThreatenedBy>>	Represents asset is under threat from attack or adversary action.

Mathematically, a general SWRL rule infers relationships based on classes and relationships within an ontology 𝒪=(𝒞,ℛ,𝒜), where 𝒞, ℛ, and 𝒜 represent classes, relationships, and axioms within the ontology, respectively. The structure of the rule can be given as:

$$C_{1}(c_{1})\wedge C_{2}(c_{2})\wedge \ldots \wedge C_{n}(c_{n})\;\wedge R_{1}(c_{i},c_{j})\wedge \ldots \wedge R_{m}(c_{p},c_{q})\;⟹R_{1}^{\prime }(c_{u}^{\prime },c_{v}^{\prime })\wedge R_{2}^{\prime }(c_{w}^{\prime },c_{z}^{\prime })\wedge \ldots$$ (2)

- $C_{i},C_{j}^{\prime }\in 𝒞$ represent class from the set of classes 𝒞.
- $R_{i},R_{j}^{\prime }\in ℛ$ represent relationship from the set of relationships ℛ.
- $c_{1},c_{2},\ldots ,c_{n}^{\prime }$ are user-defined variables for classes.

This generalized form illustrates that SWRL rules are constructed over the ontology schema by specifying ontology classes and relationships. In total, 30 inference rules are designed and executed to capture adversarial attack scenarios. These rules are implemented in Protégé and processed using an automated reasoner, ensuring output that is both machine-readable and human-interpretable.

To evaluate the correctness and feasibility of these rules, we perform a quantitative assessment in terms of the coverage of the rules and logical consistency. Furthermore, computational performance is measured by recording the execution time required per rule. The evaluation focuses primarily on qualitative aspects, such as the accuracy and coverage of the rules.

### 3.5 Assessment

#### 3.5.1 Risk assessment.

Risk assessment systematically identifies, analyzes, and manages risks associated with a system [49]. The core of this assessment involves calculating risk by evaluating two key factors: *likelihood* and *impact*, based on identified security threats [39]. The likelihood measures the probability that a threat will successfully exploit a vulnerability in the AI system. It can be assessed using threat scenarios, historical data, and expert judgment. Impact, on the other hand, refers to the potential consequences of successful threat exploitation, including financial loss, reputational damage, and operational disruption.

The risk assessment frequently employs a 3x3 risk matrix [50] and is classified into high, medium, and low categories, as shown in Table 7. These categories can also be represented by scores of 3, 2, and 1, respectively, which serve as a guide for the urgency of mitigation efforts [50]. To calculate risk, we used the classic formula [39]:

$$R(T_{i})=I_{i}\times L_{i}\;$$ (3)

**Table 7:** Risk assessment.

Likelihood	Impact	Severity Level
Low	Low	Low
Low	Medium	Low
Low	High	Medium
Medium	Low	Low
Medium	Medium	Medium
Medium	High	High
High	Low	Medium
High	Medium	High
High	High	High

Where Li denotes the likelihood value of the i-th threat, Ii indicates the impact value of the i-th threat, and R (Ti) represents the calculated risk value for the i-th threat.

#### 3.5.2 Vulnerability assessment.

The vulnerability assessment is a crucial assessment that identifies and quantifies weaknesses within a system [49]. CVSS is a widely recognized tool for evaluating the severity of vulnerabilities. CVSS relies on mandatory base metrics that must be included in the evaluation, as shown in Table 8. Temporal and environmental metrics are optional and do not need to be considered for evaluation [51]. The resulting score, which is based on base metrics, can be translated into qualitative categories (such as none, low, medium, high, and critical), helping stakeholders effectively prioritize their vulnerability management efforts.

**Table 8:** CVSS v3.1 base metrics.

Metric	Definition	Values
Attack Vector (AV)	Reflects the context by which vulnerability exploitation is possible.	- Network (N): 1.0 (Exploitable remotely over a network) - Adjacent Network (A): 0.646 (Exploitable from an adjacent network) - Local (L): 0.395 (Exploitable with local access) - Physical (P): 0.2 (Requires physical interaction)
Attack Complexity(AC)	Describes the conditions beyond the attacker’s control that must exist to exploit the vulnerability.	- Low (L): 0.77 (No special conditions) - High (H): 0.44 (Special conditions required)
Privileges Required (PR)	Indicates the level of privileges an attacker must possess before successfully exploiting the vulnerability.	- None (N): 0.85 (No privileges required) - Low (L): 0.62 (Basic user capabilities required) - High (H): 0.27 (Significant control required)
User Interaction (UI)	Captures the requirement for user participation in the successful exploitation of the vulnerability.	- None (N): 0.85 (No user interaction) - Required (R): 0.62 (User interaction required)
Scope (S)	Reflects the impact of the vulnerability on other components beyond the vulnerable component.	- Unchanged (U): 1.0 (Affects only resources managed by the same authority) - Changed (C): 1.0 (Affects resources beyond the authority of the vulnerable component)
Confidentiality (C)	Measures the impact on the confidentiality of the information resources due to a successful exploit.	- None (N): 0.0 (No impact) - Low (L): 0.22 (Some impact, limited access) - High (H): 0.56 (Significant impact, sensitive information accessible)
Integrity (I)	Measures the impact on the Integrity of the information resources due to a successful exploit.	- None (N): 0.0 (No impact) - Low (L): 0.22 (Some impact, limited modifications) - High (H): 0.56 (Significant impact, extensive modifications)
Availability (A)	Measures the impact on the availability of the information resources due to a successful exploit.	- None (N): 0.0 (No impact) - Low (L): 0.22 (Some impact, temporary interruptions) - High (H): 0.56 (Significant impact, extended unavailability)

Using the Exploitability and Impact scores, we calculate the final base score. The Exploitability score reflects how easily a vulnerability can be exploited, while the impact score measures the potential damage caused by a successful exploit. To calculate the score, the following formulas are used, which are based on the CVSS v3.1 metrics. The final rating score can be shown in Table 9.

1. **Exploitability Score:** The Exploitability score is calculated using the formula:
  Exploitability=8.22×AV×AC×PR×UI (4)
2. **Impact Score:** The Impact score depends on whether the Scope (S) is unchanged (U) or changed (C):
  - **Unchanged Scope (S = ’U’):**
    Impact=6.42×(1−[(1−C)×(1−I)×(1−A)]) (5)
  - **Changed Scope (S = ’C’):**
    $$\text{Impact}=7.52\times (I-0.029)-3.25\times (I-0.02{)}^{15}$$ (6)
3. **Base Score:** The final Score is calculated based on the condition of Unchanged and Changed Scope:
  - **Unchanged Scope (S = ’U’):**
    Base Score=min(Exploitability+Impact,10) (7)
  - **Changed Scope (S = ’C’):**
    Base Score=min(1.08×(Exploitability+Impact),10) (8)

**Table 9:** CVSS rating.

Severity	Severity Score Range
None	0.0
Low	0.1-3.9
Medium	4.0-6.9
High	7.0-8.9
Critical	9.0-10.0

### 3.6 Automated reasoner

An automated reasoner operates on a knowledge base (which is built using an ontology) to perform key inference tasks. After operating the automated reasoner, the resulting knowledge base, enriched with inferred relationships, provides a comprehensive resource for assessing threats, vulnerabilities, mitigation strategies, and security assessments within AI-enabled systems and serves as a valuable resource for continuous monitoring and updating, adapting to the evolving threat landscape and emerging vulnerabilities.

### 3.7 Querying knowledge

The description logic (DL) can be used to retrieve knowledge using expressions based on defined relationships [52]. To identify which asset is threatened by a specific threat can be given as:

Let:

- *A* represents Threat (Individual).
- *C*_0_ represents a specific Asset (Class).
- *R* represents IsThreatenedBy (ObjectProperty).

The DL Query for individuals of the specific asset class that are threatened can be represented as:

$$\{c|c\in I(C_{0})\wedge R(c,a)\}\;$$ (9)

- {c∣...} represents the set of all individuals *c* such that the conditions following the "∣" are true.
- $c\in I(C_{0})$ means that *c* is an individual belonging to the specific Asset Class *C*_0_.
- R(c,a) means that the individual asset *c* is related to a specific threat individual *a* through the *IsThreatenedBy* property.

This query returns all individuals that are members of the specific Asset Class *C*_0_ and have an *IsThreatenedBy* relationship with the individual representing a threat.

## 4 Validation and verification

To validate the research questions, we leveraged 10 MITRE ATLAS case studies (CS) [7], as shown in Table 10, that reveal a diverse collection of attack vectors, including evasion, poisoning, and model replication. In particular, these threats are no longer limited to controlled environments but actively target production systems, underscoring the need for a comprehensive ontology that can evolve in response to emerging threat intelligence. Moreover, to mitigate potential selection bias in choosing these case studies, we ensured their relevance to AI-enabled systems and diversity in security contexts. The selection criteria included broad asset coverage, representation of diverse AI-specific attack vectors, inclusion of a wide range of known vulnerabilities, and coverage of distinct phases of the attacker tactics. This systematic approach ensured a thorough coverage of our ontology scope and effectively tested its ability to represent a variety of AI systems.

**Table 10:** Case studies.

CS-ID	Description
CS001	Evasion of deep learning detectors for malware and traffic involves crafting adversarial samples that negligibly alter data points, making them appear benign to AI models but harboring malicious intent.
CS002	Attack on Machine Translation Service by replicating models and transferring adversarial examples to exploit the target model’s weaknesses, leading to incorrect outputs or system failures in translation services.
CS003	A facial recognition system that searches public photos for matches faced a security breach due to misconfiguration, allowing unauthorized access and risking misclassification in its deployed models.
CS004	Using GPT-2 Model Replication to cause excessive computational resource consumption, effectively slowing down the system or crashing it by overwhelming AI computations with complex inputs.
CS005	Tay Poisoning involves compromising the integrity of the AI model during its development stage, inserting malicious data into the training process which is then passed on to users, compromising security.
CS006	Backdoor Attack on Deep Learning Models in Mobile Apps involves embedding hidden behaviors or triggers in mobile app models, allowing remote access to the system or data.
CS007	Arbitrary Code Execution with Google Colab involves exploiting inputs that interact with the AI system, specifically in a shared environment, allowing an attacker to execute arbitrary code on the host machine.
CS008	PoisonGPT involves manipulating the training process or data of a Generative Pre-trained Transformer to generate biased or malicious content, misleading users or automating the spread of disinformation.
CS009	Indirect Prompt Injection Threats: Bing Chat Data Pirate involves injecting crafted inputs or prompts into AI systems, specifically in natural language processing contexts, misleading the AI to generate harmful responses.
CS0010	ChatGPT Plugin Privacy Leak occurs when sensitive information is inadvertently included in the AI’s training data or outputs, exposing personal data to unauthorized parties and violating privacy and compliance regulations.

To answer *RQ1*, this study investigates the effectiveness of a designed ontology in addressing the challenges of inconsistent or insufficient AI systems representation. By developing a formal data representation and automating the threat identification process, this RQ aims to enhance the security analysis capabilities across the evolving AI threat landscape. To evaluate, the provided case studies serve as a foundation to evaluate how ontology can encapsulate and integrate data within a unified knowledge base.

To begin with, the ontologies are crafted to provide a *general representation* of AI assets. These ontologies include classes and subclasses that standardize the description of various AI assets across systems, ensuring unified representation. The representation of the identified assets across all cases from ontology is outlined as follows in Table 11. These classes are structured hierarchically to reflect their interrelationships within the system. For example, a Deep Learning Detector is modelled as a sub-asset of a Security Mechanism, which in turn is a sub-asset of an Asset. Consequently, the Deep Learning Detector is classified as a subsub-asset of an Asset. This organization facilitates a clear and consistent representation of the relationships among various AI system components, thereby enhancing the semantic clarity of the ontology.

**Table 11:** Hierarchical representation of assets across case studies.

CS-ID	Identified SubSub-Asset	Sub-Asset	Asset
CS001	Deep Learning Detector	Deep Learning Detector SubClassOf Security Mechanism	Security Mechanism SubClassOf Asset
CS002	Machine Translation Service	Machine Translation Service Type Software	Software SubClassOf Asset
CS003	Facial Recognition System	Facial Recognition System Type Software	Software SubClassOf Asset
CS004	Cloud Computing Resource	Cloud Computing Resource Type Resource	Resource SubClassOf Asset
CS005	Chatbot	Chatbot SubClassOf Tool	Tool SubClassOf Asset
CS006	Mobile Application & Deep Learning Model	Mobile Application Type Software & Deep learning Detector SubClassOf Security Mechanism	Software SubClassOf Asset & Security Mechanism SubClassOf Asset
CS007	Collaborative Platform	Collaborative Platform Type Software & Collaborative Platform Type Platform	Software SubClassOf Assets & Platform SubClassOf Asset
CS008	GPT	GPT Type LLM & LLM SubClassOf Model Model Type Software	Model SubClassOf Asset
CS009	Chat System	Chat System SubClassOf Tool	Tool SubClassOf Asset
CS010	Chat System	Chat System SubClassOf Tool	Tool SubClassOf Asset

Building upon the foundational representation, the ontology establishes *relationships* that describe interactions between entities, covering a broad spectrum of interactions. For example, in the case of *"CS001"*, we discover a threat named "Detection Evasion", which provides broad information through various relationships, as follows:

- **Name:** Detection Evasion
- **Type:** Security Threat
- **Has:** High Risk
- **Description:** This type of threat involves techniques designed to evade detection from AI-powered security systems. Attackers may use methods that manipulate data inputs in a way that causes the AI model to misclassify or overlook malicious activities. This can include the use of adversarial examples that subtly alter data points so they appear benign to AI models but are malicious.
- **IsMitigatedBy:**
  1. AML.M0003 (Model Hardening)
  2. AML.M0015 (Adversarial Input Detection)
  3. OWASP.M0019 (Input Validation)
- **Exploits:**
  1. Training Data Poisoning
  2. Improper Input Validation
  3. Deserialization of Untrusted Data
- **Violates:** Integrity, Confidentiality
- **IsInitiatedBy:** Bots
- **Individual:** AML.A0001
- **Techniques LeadsTo Threat:**
  1. AML.T0000 → AML.A0001
  2. AML.T0006 → AML.A0001
  3. AML.T0015 → AML.A0001
  4. AML.T0043 → AML.A0001

The description of this threat highlights its capability to define risk and suggest mitigation measures. It also identifies which weaknesses are potentially exploited and which properties are violated by this threat. Additionally, it identifies the adversarial techniques that could contribute to this threat. Thus, ontology facilitates the description of entities and their relationships with other entities to provide complete information that guides the development of effective countermeasures, ensuring the security of AI systems.

Moreover, to enhance the *inferential capabilities*, the ontology incorporates inference rules to enforce logical consistency across AI systems. SWRL specifies logical rules using threat scenarios to identify potential threats to at-risk assets and facilitate mitigation strategies. For instance, in the case of *CS002*, we outline the scenario and apply the rule, which assists in identifying threats to a machine translation system’s model, as detailed as:

**Rule Scenario:** If a model is replicated for an adversarial attack, then this model is threatened by a model replication attack.

**SWRL Rule:** Model(?M), IsReplicatedBy(?M, ?A), AMLA0004(?T), Adversary(?A) -> IsThreatenedBy(?M, ?T).

**Rule Mapping:** Replication Attack to Model

Furthermore, to ensure a *holistic perspective*, the ontology integrates the stages of the *AI lifecycle*, including data understanding, data preparation, model engineering, model evaluation, deployment, monitoring, and maintenance. This coverage ensures that the standardization process encompasses the entire AI lifecycle, facilitating the integration of a vast number of mitigation mechanisms and updates. For example, the following stages incorporate several mitigation strategies that can be applied in response to emerging threats and vulnerabilities.

- 06 Mitigations *AreIncludedIn* Business and Data Understanding
- 08 Mitigations *AreIncludedIn* Data Preparation
- 17 Mitigations *AreIncludedIn* Model Engineering
- 14 Mitigations *AreIncludedIn* Model Evaluation
- 15 Mitigations *AreIncludedIn* Deployment
- 14 Mitigations *AreIncludedIn* Monitoring and Maintenance

Complementarily, to provide a broader context, the ontology also integrates the *tactics and techniques* used by adversaries, providing a more general view of the security landscape; for example, in case studies, various tactics and techniques are used by the adversary, as shown in Table 12.

**Table 12:** Tactics and techniques used across case studies.

Tactic	Technique	CS-ID
Adversary Uses Attack Staging	Attack Staging ConsistOf AML.T0005 (Create Proxy ML Model)	CS002
Adversary Uses Defense Evasion	Defense Evasion ConsistOf AML.T0015 (Evade ML Model)	CS001
Adversary Uses Execution	Execution ConsistOf AML.T0050 (Command and Scripting Interpreter)	CS007
Adversary Uses Exfiltration	Exfiltration ConsistOf AML.T0057 (Data Leakage)	CS010
Adversary Uses Impact	Impact ConsistOf AML.T0020 (Poison Training Data)	CS008
	Impact ConsistOf AML.T0029 (Denial of ML Service)	CS004
	Impact ConsistOf AML.T0046 (Spamming ML System with Chaff Data)	CS004
Adversary Uses Initial Access	Initial Access ConsistOf AML.T0003 (Search Victim-Owned Websites)	CS003
	Initial Access ConsistOf AML.T0004 (Search Application Repositories)	CS003
	Initial Access ConsistOf AML.T0029 (Supply Chain Compromise)	CS005
Adversary Uses Persistence	Persistence ConsistOf AML.T0018 (Backdoor ML Model)	CS006
	Persistence ConsistOf AML.T0051 (Prompt Injection)	CS009

Ultimately, enabling practical application, the ontology can be queried to *extract information*. DL queries are a powerful way to retrieve specific knowledge that is explicitly stated or logically implied within the ontology. Hence, the ontology supports a wide range of such information retrieval requests, enabling users to explore and understand the relationships and characteristics of the concepts (like assets and threats) defined within it.

- **Query:** Assets and (*IsThreatenedBy* value AML.A0022)
- **Query Results:**
  1. Training Data
  2. Validation Data
  3. Digital Data

To *conclude*, our ontology-driven approach may help address domain-specific challenges by establishing a formal vocabulary. Although not yet a widespread or standardized vocabulary, it offers a shared knowledge base that can support consistent understanding and implementation of AI security measures across different systems. This knowledge base also has the potential to facilitate collaboration among various stakeholders.

**RQ**_**1**_ **Summary:** *Our findings indicate that the ontology supports a unified representation of knowledge and provides broader coverage of AI-specific threat modelling aspects compared to existing approaches. In particular, the ontology captures (1) AI-specific assets, (2) weaknesses, (3) security threats, and their (4) potential mitigations, (5) AI lifecycle stages, and (6) adversarial tactics and techniques in a single knowledge base, while also linking them to risk and vulnerability assessments. This integrated view enables reasoning across different dimensions of AI security that, to our knowledge, are not jointly addressed in prior research.*

To answer *RQ2*, we evaluated the effectiveness of the inference rules in accurately identifying potential threats to system assets across various AI systems through the case studies. These rules are crafted to detect a wide array of security threats to AI systems by automating the threat identification process.

To begin with, by *automating threat identification*, these rules demonstrate the capability to uncover vulnerabilities that might otherwise remain undetected in complex AI environments. Table 13 summarizes various potential threats to AI systems and the corresponding assets affected, the potential mitigations suggested, and the vulnerabilities that can be potentially exploited by the threats are outlined.

**Table 13:** AI system security: Threats, assets, countermeasures and vulnerabilities.

CS-ID	Threat Identified	Asset Affected	Mitigation Needed	Vulnerability Identified
CS0001	Detection Evasion	Deep Learning Detector	1. Model Hardening 2. Adversarial Input Detection 3. Validate ML Model	CWE-20: Improper Input Validation LLM03: Training Data Poisoning
CS0002	Replication Attack	Machine Translation Service	1. Model Obfuscation	CWE-125: Out-of-bounds Read
CS0003	Misconfiguration of Source File	Facial Recognition System	1. Verify ML Artifacts	CWE-16: Misconfiguration CWE-284: Improper Access Control
CS0004	Denial of Services	Cloud Computing Resources	1. Restrict the Number of Queries 2. Implement resource isolation techniques.	LLM04: Model Denial of Service CWE-787: Out-of-bounds Write
CS0005	Supply Chain Poisoning	AI Chatbots	1. Input Validation 2. Sanitize Training Data	LLM03: Training Data Poisoning CWE-20: Improper Input Validation.
CS0006	Backdoor Attack	Mobile Application, Deep Learning Models	1. Model Hardening	CWE-693: Protection Mechanism Failure.
CS0007	Arbitrary Code Execution	Collaborative Platforms	1. Code Signing 2. Vulnerability Scanning	CWE-94: Improper Control of Generation of Code (’Code Injection’) CWE-502: Deserialization of Untrusted Data
CS0008	GPT Poisoning	Generative AI Models (GPT)	1. Validate ML Models 2. Sanitize Training Data	CWE-20: Improper Input Validation LLM03: Training Data Poisoning
CS0009	Prompt Injection	AI-powered Chat Systems	1. Model Hardening 2. Adversarial Input Detection 3. Input Validation	LLM01: Prompt Injection Vulnerability CWE-20: Improper Input Validation
CS0010	Privacy Leak	AI Chat Systems	1. Encrypt Sensitive Information	LLM06: Sensitive Information Disclosure CWE-125: Type Out-of-bounds Read

For instance, in the case of *CS0001*, the threat labeled *Detection Evasion* targets the Deep Learning Detector, a critical asset responsible for identifying malicious or adversarial behaviors. This threat involves attempts by adversaries to bypass detection mechanisms, often by exploiting weaknesses within AI models. To mitigate such risks, several measures are recommended: (1) Model Hardening, which involves strengthening the model against adversarial attacks through techniques such as adversarial training; (2) Adversarial Input Detection, to identify and filter potentially malicious inputs before they affect model performance; and (3) Validation of Machine Learning Models, ensuring regular evaluation to detect vulnerabilities. The identified vulnerabilities associated with this threat include CWE-20: Improper Input Validation, where unvalidated inputs allow system manipulation, and LLM03: Training Data Poisoning, where adversaries inject malicious samples into training datasets to corrupt the model’s behavior.

In addition, the rules are effective in maintaining a high level of *accuracy*. For example, rules targeting specific threats like detection evasion are not only accurate in their identification but also adaptable to various AI system components, such as chatbots, facial recognition systems, and authentication mechanisms.

Finally, the rules identify which *mitigation measures* for the respective threats can be incorporated at their respective lifecycle stages. This ensures that security is integrated throughout the AI system’s development process. For example, during the Model Engineering Phase, we can incorporate the mitigation measures such as:

- Model Hardening
- Model Obfuscation
- Adversarial Input Detection

In *general*, inference rules effectively uncover *threats* and *vulnerabilities* with high accuracy. Their integration across lifecycle stages enhances proactive security, validating their role in strengthening AI threat modelling. The results demonstrated consistency and accuracy in mapping threats and vulnerabilities to their respective entities of the AI system. Importantly, the rules enabled proactive identification of risks before deployment, highlighting their usefulness in early-stage threat modelling. From a *performance perspective*, the reasoning achieved 100% logical consistency with no redundant or contradictory outputs, and each rule executed in less than two seconds, demonstrating computational feasibility. Hence, the current validation primarily across case studies emphasizes qualitative accuracy and coverage.

**RQ**_**2**_ **Summary:** *Our findings demonstrate that the inference rules help to identify security threats to AI-enabled system assets, reveal their effectiveness in mapping out potential vulnerabilities, and corresponding mitigation strategies.*

To answer *RQ3*, to effectively conduct the *assessment* of AI-enabled systems in alignment with threat modelling, ontology reduces the likelihood of inconsistencies in the evaluation of different AI systems. For example, when assessing the risks of identified threats, an ontology-based approach allows the calculation of key risk factors, such as likelihood and impact, as illustrated in Table 14 in all cases. This ensures that the risk assessment process remains consistent, regardless of who assesses the AI system. The final risk score represents the severity of a threat: a score of 6 indicates high severity, whereas a score of 4 denotes medium severity. For example:

- CS0001 - Detection Evasion *Has* High Severity
- CS0008 - Prompt Injection *Has* Medium Severity

**Table 14:** Assessment for each identified threat across case studies.

CS-ID	Threat Name	Impact	Likelihood	Risk Score
CS0001	Detection Evasion	3	2	6
CS0002	Replication Attack	3	2	6
CS0003	Misconfiguration of Source file	3	2	6
CS0004	Denial of Services	3	2	6
CS0005	GPT Poisoning	3	2	6
CS0006	Backdoor Attack	3	2	6
CS0007	Supply Chain Poisoning	3	2	6
CS0008	Prompt Injection	2	2	4
CS0009	Arbitrary Code Execution	3	2	6
CS0010	Privacy Leak	3	2	6

Similarly, the *severity* of each system weakness is assessed using the CVSS metrics, which assigns a numerical score ranging from 0 to 10 to quantify the severity level of each identified weakness. A higher score indicates greater severity and urgency, while a lower score reflects a less critical weakness. In the case studies, we identified the potential weaknesses along with their severity scores, as shown in Table 15. This scoring helps prioritize which vulnerabilities require immediate attention and remediation. For example, CWE-20 (Improper Input Validation) is rated with the highest severity (score 10) in 4 case studies, implying it is a critical and common weakness that can lead to serious exploitation if not addressed. LLM03 (Training Data Poisoning), with a high score (7.4) in 3 case studies, shows that data integrity attacks in large language models are also a major concern, particularly for AI models relying heavily on external data.

**Table 15:** Assessment for each identified weakness across case studies.

CS-ID	Weakness	Score
CS0009	LLM01: Prompt Injection	9.6
CS0001, CS0005, CS0008	LLM03: Training Data Poisoning	7.4
CS0004	LLM04: Model Denial of Service	8.6
CS0010	LLM06: Sensitive Information Disclosure	9.9
CS0001, CS0005, CS0008, CS0009	CWE-20: Improper Input Validation	10.0
CS0002, CS0010	CWE-125: Out-of-bounds Read	9.9
CS0003	CWE-16: Misconfiguration	7.4
CS0003	CWE-284: Improper Access Control	7.1
CS0004	CWE-787: Out-of-bounds Write	7.5
CS0006	CWE-693: Protection Mechanism Failure	7.4
CS0007	CWE-94: Code Injection	9.9
CS0007	CWE-502: Deserialization of Untrusted Data	9.9

In *general*, the findings underscore the need to implement targeted mitigation strategies for both high-risk threats and high-severity vulnerabilities. The findings also emphasize the effectiveness of using risk and severity-based scoring systems in systematically prioritizing security efforts.

**RQ**_**3**_ **Summary:** *Our findings reveal that the ontology-driven method provides a structured and semantically consistent approach to risk and vulnerability assessment in AI-enabled systems. Unlike previous approaches, which often rely on manual evaluations or lack assessment mechanisms, our method enhances consistency among stakeholders and delivers assessment benefits aligned with established threat-modelling practices.*

To verify the *correctness and completeness* of ontology-driven threat modelling, we adopt a theorem-based approach to formally validate the transformation process [53,54]. In this context, transformation refers to the formal mapping of ontological elements from their initial representation into their updated representation after the application of inference rules and reasoning mechanisms. This validation ensures that, before and after the automated reasoner’s execution, the transformation preserves the logical structure of the ontology. Specifically, it guarantees that the integrity of *relationships, classes, and objects* within the ontology, i.e., the logical connections among these elements, remains consistent and is not disrupted throughout the reasoning process. Here, *correctness* ensures that the inference rules and reasoning steps do not introduce contradictions or invalid relationships in the ontology. In contrast, *completeness* ensures that all classes, objects, and relationships derivable from the ontology are indeed captured and represented during the reasoning process.

### Correctness of ontology-driven approach

Ensuring the *correctness* of an ontology-driven solution requires that the transformation preserve logical consistency and semantic soundness. As established in prior ontology engineering studies [55,56], correctness can be demonstrated through structural induction, where transformation maintains the validity of the ontology’s axioms and relationships.

To verify correctness, consider classes *S*, objects *a*, and relationships *c*. The correctness can be proved by demonstrating the truth of the following expression:

$$\text{If }T(S,a,c)=O^{\prime \prime }\text{ then }(S,a,c{)}_{\text{before}}\to (S^{\prime \prime },a^{\prime },c^{\prime }{)}_{\text{after}}\;$$ (10)

Here, *O* represents the ontology, which consists of classes, objects, and relationships; $(S,a,c{)}_{\text{before}}$ represents the ontology before transformation (*T*), and $(S^{\prime \prime },a^{\prime },c^{\prime }{)}_{\text{after}}$ represents the ontology after transformation (*T*).

#### Base case.

In the base case, we establish the conditions for the transformation process:

- *S* = *S*: The component *S* in its initial state remains unchanged before transformation.
- *a* = *a*: Similarly, the component *a* remains unchanged before the transformation.
- *c* = *c*: Likewise, the component *c* remains unchanged before the transformation.

These statements emphasize that no change has occurred yet; each component is in its original state. Using these conditions, the base case for performing the transformation is represented as:

$$(S,a,c{)}_{\text{before}}\to (S,a,c{)}_{\text{after}}$$ (11)

The base case implies that the ontology (*O*) along with its components, *S*, *a*, and *c* undergoes a process that changes their state from "before" to "after," but these remain consistent and the same before and after the transformation.

#### Induction hypothesis.

To extend the proof, we use an induction hypothesis. Assume: $a=a^{\prime }\text{and}c=c^{\prime }$. Substituting these into expression (10) gives us expression (12):

$$\text{If }T(S,a^{\prime },c^{\prime })=O^{\prime \prime }\text{ then }(S,a^{\prime },c^{\prime }{)}_{\text{before}}\to (S^{\prime \prime },a^{\prime },c^{\prime }{)}_{\text{after}}\;$$ (12)

To further verify the correctness, we transform the ontologies along with their classes, objects, and relationships. After the transformation process, the ontology equation becomes:

$$(S,a,c{)}_{\text{before}}\to (S^{\prime },a^{\prime },c^{\prime }{)}_{\text{after}}\;$$ (13)

By applying the induction hypothesis, the state of the ontology after the transformation process becomes:

$$(S^{\prime },a^{\prime },c^{\prime }{)}_{\text{after}}\to (S^{\prime \prime },a^{\prime },c^{\prime }{)}_{\text{after}}\;$$ (14)

By applying the transitivity relation between steps (13) and (14), the resulting transformation is:

$$(S,a,c{)}_{\text{before}}\to (S^{\prime \prime },a^{\prime },c^{\prime }{)}_{\text{after}}\;$$ (15)

Hence, the final equation (15) proves expression (10), confirming that the ontology-driven approach is correct. The equation demonstrates the accurate transformation process from $(S,a,c{)}_{\text{before}}$ to $(S^{\prime \prime },a^{\prime },c^{\prime }{)}_{\text{after}}$.

### Completeness of ontological-driven approach

In addition to correctness, *completeness* ensures that all relevant entities and relationships within the domain are representable and inferable through the ontology. Previous works [56,57] define completeness as the preservation of structural integrity and inferential coverage after transformation. Building on this, our verification process demonstrates that every defined entity, relationship, and rule within OntoSecAI-DO remains intact and inferable following reasoning.

To verify completeness, the proof should demonstrate the preservation of structural integrity within the scope of the defined ontology and its associated inference rules. In this context, equation (16) confirms that all entities and relationships specified in the ontology are preserved throughout the transformation process. This preservation is guaranteed under the assumption of *correctness*, as formally established in equation (15).

$$\text{If }T(S,a,c{)}_{\text{before}}\to (S^{\prime \prime },a^{\prime },c^{\prime }{)}_{\text{after}}\text{ then }(S^{\prime \prime },a^{\prime },c^{\prime })=O^{\prime \prime }\;$$ (16)

In this expression:

- $(S,a,c{)}_{\text{before}}$ represents the state of the ontology before transformation.
- $(S^{\prime \prime },a^{\prime },c^{\prime }{)}_{\text{after}}$ represents the state of the ontology after transformation.
- $(S^{\prime \prime },a^{\prime },c^{\prime })=O^{\prime \prime }$ denotes the complete ontology or knowledge base.

#### Base case.

For the base case, we start by considering the basic transformation, which can be represented as follows:

$$(S,a,c{)}_{\text{before}}\to (S,a^{\prime },c^{\prime }{)}_{\text{after}}$$ (17)

Here, we know that the initial state $(S,a,c{)}_{\text{before}}$ is equivalent to the ontology *O*. Therefore, the expression simplifies to:

$$T(S,a^{\prime },c^{\prime }{)}_{\text{after}}=O$$ (18)

This base case shows that the transformation retains the integrity of the ontology’s basic structure.

#### Induction hypothesis.

To extend this proof through induction, we assume that the hypothesis holds for the transformation. Specifically, let $a=a^{\prime }$ and $c=c^{\prime }$. Under this assumption, expression (16) from the previous section becomes:

$$\text{If }T(S,a^{\prime },c^{\prime }{)}_{\text{before}}\to (S^{\prime \prime },a^{\prime },c^{\prime }{)}_{\text{after}}\text{ then }T(S^{\prime \prime },a^{\prime },c^{\prime })=O^{\prime \prime }\;$$ (19)

This expression represents the transformation process leading to the complete ontology. Within this ontology, the classes (*S*), objects (*a*), and relationships (*c*) constitute the core elements of the ontology (*O*), such that S,a,c∈O. The transformation process can be summarized by the following expression:

$$T(S,a,c)=T(S^{\prime },a^{\prime },c^{\prime })\;$$ (20)

We know that when a transformation is applied, the resulting ontological state $T(S^{\prime },a^{\prime },c^{\prime })$ is equivalent to an ontological state represented as $T(S^{\prime \prime },a^{\prime },c^{\prime }{)}_{\text{after}}$:

$$T(S^{\prime },a^{\prime },c^{\prime })=T(S^{\prime \prime },a^{\prime },c^{\prime }{)}_{\text{after}}\;$$ (21)

Furthermore, based on the formal definition of completeness, we assert that:

$$T(S^{\prime \prime },a^{\prime },c^{\prime }{)}_{\text{after}}=O^{\prime \prime }\;$$ (22)

Applying the transitivity relations of equations (21) and (22), we obtain:

$$T(S^{\prime },a^{\prime },c^{\prime })=O^{\prime \prime }\;$$ (23)

The expression (23) shows that after transformation, the ontology consists of all classes, objects, and relationships. Further, given the expressions (20) and (23) and their transitive relations, we conclude that:

$$T(S,a,c)=O^{\prime \prime }$$ (24)

The final equation (24) confirms that the transformation of the ontology is complete, as it fully encompasses the structure and relationships initially present in the ontology *O*. The completeness of our framework $O^{\prime \prime }$ is thus proven in terms of ensuring that all necessary components of the ontology are preserved and completely transformed through each stage of the process.

## 5 Threats to validity

This section recognizes and addresses potential threats to the robustness of the proposed approach. By recognizing and mitigating these threats, this study ensures the integrity of its methodology, from internal consistency to the generalizability and applicability of its findings.

*Threats to Internal Validity* include the influence of confounding factors on the methodology. These factors may include limitations of the tool used, ambiguities in the entities’ definition, and contextual differences among them, which could affect the accuracy of the approach. To mitigate such threats, this study used a formal vocabulary that ensures consistent and precise definitions of all relevant entities. In addition, a standard tool designed for automation is employed, which further minimizes the impact of the confounding factors. Hence, it ensures that any contextual differences do not adversely affect the accuracy of the overall approach.

*Threats to External Validity* stem from the domain-specific focus of this study. Since the research primarily targets AI-enabled systems, the generalizability of the proposed approach to other domains may be constrained. However, while the study remains AI-centric, it contributes as a foundational structure that could be adapted and extended to support security automation in other technological domains in the future.

*Threats to Construct Validity* may arise from inaccuracies in the definition and representation of ontologies, inference rules, and the assessment of risks and vulnerabilities. To mitigate this, the study populated the ontologies using a formal vocabulary carefully selected from four recognized repositories, which was then refined to support the consistent modelling of threats and vulnerabilities in AI-enabled systems. Inference rules are formally designed using SWRL, enabling a consistent and machine-interpretable representation of security logic across AI-enabled systems. Furthermore, risk and vulnerability assessments are conducted using established metrics, ensuring alignment between theoretical constructs and the study’s measures. Hence, these measures strengthen the validity of the evaluated constructs.

*Threats to Conclusion Validity* may arise if there are inaccuracies in the results obtained from case studies after validation. These inaccuracies can potentially undermine the credibility of the findings. In this study, a large number of case studies are used to demonstrate the effectiveness of the proposed approach. To further support the reliability of the conclusions drawn, the approach is also verified using mathematical theorems, ensuring both completeness and correctness. This dual validation strategy, i.e., demonstration through case studies and theoretical verification, helps in enhancing the overall reliability of the research results.

## 6 Conclusion

In this study, we presented an automated threat modelling approach that utilizes ontologies to improve AI security. This work is motivated by the fact that the widespread use of AI-enabled systems presents significant security challenges, with studies revealing their high vulnerability to various threats. Furthermore, the growing availability of AI technologies, particularly large language models like ChatGPT, Gemini, and Llama, has intensified these risks by introducing new avenues for potential threats. This presents a critical problem to existing threat modelling practices, as these new attack surfaces are often not well-documented or understood. To address these challenges, we proposed the OntoSecAI approach, in which ontologies are populated with data from well-established repositories. This practice ensures that the knowledge base provides a verifiable and accurate representation of adversarial behavior and the security posture of AI-enabled systems, a comprehensive and data-driven foundation that is essential for producing reliable threat modelling. Our findings reveal that domain ontology plays a crucial role in providing a comprehensive and consistent knowledge base for terminology and structural definitions across diverse AI systems, which in turn facilitates a shared understanding of security concepts among stakeholders and enables the implementation of more appropriate security controls. Additionally, the use of inference rules has proven highly effective in accurately identifying potential threats to system assets across various AI systems, significantly improving threat detection capabilities. By contributing to more accurate and consistent risk and vulnerability assessments, ontologies provide a well-defined approach to evaluating and managing risks and potential weaknesses in AI systems. In the future, we plan to enhance our approach by incorporating additional data sources to further enrich the ontology and by expanding the set of inference rules to capture and automate emerging threats.
